# Immune Myositis Complicating Follicular Lymphoma: Case Report

**DOI:** 10.3390/reports9010012

**Published:** 2025-12-29

**Authors:** George Sarin Zacharia, Saran Lal Ajai Mokan Dasan, Chinazor Iwuaba

**Affiliations:** Department of Internal Medicine, BronxCare Health System, New York, NY 10457, USA; sajaimok@bronxcare.org (S.L.A.M.D.); ciwuaba@bronxcare.org (C.I.)

**Keywords:** lymphoma, paraneoplastic, myopathy, myositis, antisynthetase

## Abstract

**Background and Clinical Significance**: Idiopathic inflammatory myopathies are a heterogeneous group of autoimmune disorders that may present as paraneoplastic syndromes. Although most frequently associated with solid-organ malignancies, hematological neoplasia, particularly lymphomas, is also likely linked. **Case Presentation**: We describe a sexagenarian female with progressive proximal muscle weakness, myalgias, and lymphadenopathy. Laboratory evaluation revealed markedly elevated creatine phosphokinase and myositis-specific antibodies: anti-Mi-2α and anti-EJ. Magnetic resonance imaging of the thighs confirmed active myositis. Lymph node biopsy reported follicular lymphoma. The patient was initiated on methotrexate and rituximab, with which she reported significant symptomatic relief. **Conclusions**: Inflammatory myopathy is an exceedingly rare presentation of follicular lymphoma. This case emphasizes that lymphomas can closely mimic other disease processes and present significant diagnostic challenges, and they should be included in the differential diagnosis of myopathies. Improved awareness and early diagnosis of lymphoproliferative neoplasia often yield better overall clinical outcomes.

## 1. Introduction and Clinical Significance

Idiopathic inflammatory myopathies (IIM) are rare immune-mediated disorders with skeletal muscle inflammation and variable extramuscular involvement. Dermatomyositis and polymyositis are the classical subtypes; others include immune-mediated necrotizing myopathy, antisynthetase syndrome, and inclusion body myositis. Intriguingly, one-fourth of patients with adult-onset IIM are diagnosed with cancer within three years of diagnosis of IIM [1]. The link between myopathy and cancer likely involves shared autoantigens and dysregulated immune responses. Solid tumors, including ovarian, lung, and gastrointestinal cancers, are most frequently implicated. Hematological malignancies are less often associated with IIM. A retrospective study by Marie et al. identified 32 cases of dermatomyositis (DM) or polymyositis (PM) associated with hematological neoplasia over 5 years across 10 institutions; only one patient had follicular lymphoma [2]. The association of follicular lymphoma with IIM is exceedingly rare, with only a handful of cases published in the medical literature to date [2,3]. Here, we report a case of adult-onset inflammatory myopathy associated with follicular lymphoma, to highlight an uncommon clinical presentation and add to the published literature on the association between these pathological entities.

Clinical Significance: Adult-onset inflammatory myopathies are often paraneoplastic and are frequently associated with solid-organ neoplasia, whereas hematological malignancies are less common culprits. This case of inflammatory myopathy associated with follicular lymphoma is a rarity. Still, it highlights that lymphomas are ‘great masquerades’ and should be excluded in virtually all clinical presentations, especially when the etiology is unclear.

## 2. Case Presentation

A 61-year-old African American female presented with progressive proximal muscle weakness, generalized myalgias, and dysphagia to both solids and liquids of 2 months duration. She reported difficulty performing daily tasks such as combing her hair and rising from a seated position. Also, she noticed a weight loss of approximately 15 pounds over the same period. She denied fever, rash, Raynaud’s phenomenon, or arthralgia. Her past medical history was significant for hypertension, uterine fibroids, and diabetes mellitus.

Physical examination revealed proximal muscle weakness: grade 3/5 in the upper extremities and grade 4/5 in the lower extremities, respectively. There was no distal muscle weakness or sensory loss. She had palpable non-tender inguinal lymphadenopathy. No cutaneous lesions were identified, and the remainder of the systemic examination was unremarkable.

Laboratory investigations revealed significantly elevated creatine phosphokinase (10,510 IU/L), aspartate aminotransferase (400 IU/L), lactate dehydrogenase (912 IU/L), and aldolase (68.1 IU/L) levels. The baseline hematological and biochemical parameters are summarized in Table 1. She was positive for antinuclear antibody (ANA) with a titer of 1:1280, with a homogeneous/speckled pattern. The myositis-specific antibody panel was notable for the presence of anti-Mi-2α and anti-EJ antibodies, suggesting an inflammatory myopathy. Other autoimmune markers, including anti-dsDNA, anti-RNP, SSA/SSB, and Scl-70, were non-reactive.

**Figure 1:**
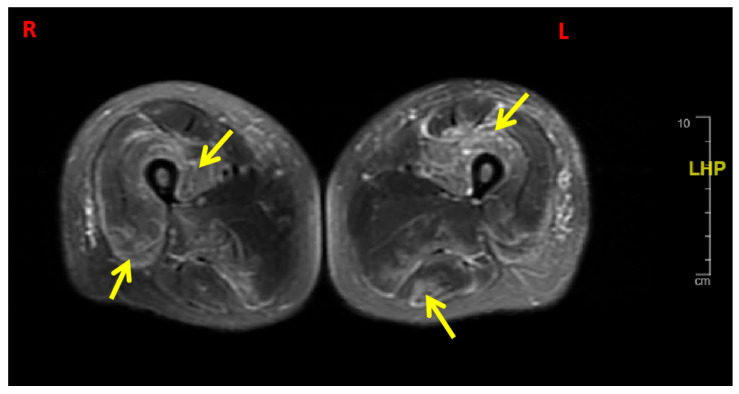
Magnetic resonance imaging of the right (R) and left (L) thighs, T2-weighted axial images. Images reveal diffuse hyperintensity and edema involving the quadriceps and hamstrings, consistent with active myositis.

Magnetic resonance imaging (MRI) with contrast of the thighs, performed on day 4 of initial presentation, revealed diffuse, symmetric muscle edema and enhancement throughout all compartments, which is consistent with active myositis (Figure 1). Abdominal ultrasound imaged fatty hepatomegaly, cholelithiasis, and multiple masses in the midline abdomen, which is most consistent with lymphadenopathy. Computed tomography of the chest, mammography, and echocardiography were within normal limits. The electromyography on day 16 was reported as short-duration, short-amplitude muscle unit action potentials and spontaneous fibrillation potentials, consistent with inflammatory myopathy.

**Figure 2:**
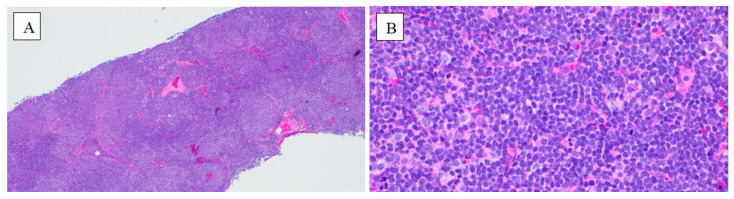
Lymph node histopathology. (**A**) Demonstrates effaced nodal architecture with atypical follicular nodularity and ill-defined margins (Stain: Hematoxylin and Eosin; Magnification: 100×). (**B**) Neoplastic follicles composed predominantly of centrocytes (germinal center B cells), consistent with follicular lymphoma (Stain: Hematoxylin and Eosin; Magnification: 400×).

A core biopsy of a right groin lymph node, performed on day 10 and reported on day 28 of initial presentation, confirmed follicular lymphoma, grade 1–2 of 3, with immunohistochemistry showing positivity for CD20, CD10, BCL-2, BCL-6, and PAX-5. The Ki-67 proliferation index was low (1–2+ out of 4+). Flow cytometry of peripheral blood and lymph node aspirate identified a monoclonal B-cell population (9%) that expresses CD19, CD20, CD10, and CD200, and lacks CD5 and CD23, which is consistent with a follicle center B-cell lymphoproliferative disorder. (Figure 2). Positron emission tomography (PET) demonstrated extensive hypermetabolic lymphadenopathy in the cervical, thoracic, abdominal, and pelvic regions, along with diffuse splenic involvement. A muscle biopsy was deferred as the patient opted against it.

The diagnosis of inflammatory myopathy associated with follicular lymphoma (Ann Arbor stage III; FLIPI score 5) was made, though it was limited by the absence of a muscle biopsy. The patient was initiated on methotrexate 15 mg weekly with folic acid supplementation once the diagnosis was made (day 18), resulting in significant symptomatic relief and a marked reduction in CPK levels. With regard to management of lymphoma, the oncology team, after extensive discussion with the patient, opted for rituximab monotherapy, as she declined cytotoxic chemotherapy. She was planned for four weekly doses followed by maintenance therapy. She received the first dose of rituximab almost 2.5 months after the initial presentation, at a dose of 375 mg/m^2^. The initiation of treatment was delayed as the patient was very hesitant to initiate any form of cancer therapy. She completed three doses of rituxumab and was subsequently lost to follow-up. At the last visit, with our institution, the patient continued under joint care by the rheumatology and oncology teams. As per clinic records, her symptoms resolved, and she was able to perform day-to-day activities independently. The serial laboratory parameters are summarized in Table 1.

## 3. Discussion

IIM, often referred to as myositis, is a heterogeneous family of autoimmune diseases that affects not only skeletal muscle but also other organ systems, such as the skin, joints, and lungs [4]. The estimated annual incidence and prevalence of IIM are 0.2–2 and 2–25 per 100,000, respectively [5]. They include DM, PM, immune-mediated necrotizing myopathy (IMNM), and inclusion body myositis (IBM). DM is the most common variant, while PM is the least common; IBM is the most common myopathy in individuals over the age of 50 years [6]. Genetic influences, molecular mimicry, and viral triggers play a potential role in the pathogenesis of IIM [7,8].

DM/PM presents with progressive, symmetric proximal muscle weakness developing over weeks to months. Distal weakness occurs and develops later in the course of the disease. Patients struggle with activities such as climbing stairs, rising from a chair, or combing hair. Facial and extraocular muscles are typically spared, while patients could have dysphagia [9]. DM is characterized by skin rashes such as heliotrope rash, Gottron’s papules, V-sign, and shawl sign. DM variants include amyotrophic DM, skin disease without overt muscle weakness, which still shows subclinical muscle involvement on biopsy, and ‘DM sine dermatitis’ with isolated muscle involvement. Contrary to DM/PM, IBM is frequently associated with early-onset distal weakness [6]. Clinical features of concomitant autoimmune diseases or underlying neoplasia may overlap with the clinical features of myopathy.

Diagnosis relies on markers of muscle injury in the presence of autoantibodies, supported by imaging features of myositis, electromyography (EMG), and biopsy. The muscle biopsy remains the gold standard for diagnosis and demonstrates inflammatory infiltrations. The serum markers of muscle injury include CPK, aminotransferases, lactate dehydrogenase, and aldolase. A myriad of autoantibodies have been described in IIM and cancer-associated myopathies; specific assays would help in diagnosis. Table 2 summarizes the types of autoantibodies and corresponding inflammatory myopathies. A large-scale study by Kerola et al., including more than 1000 subjects with positive myositis antibodies, found that anti-HMGCR antibodies had the highest positive predictive value (94%) for myositis. Antisynthetase antibodies had the highest positive predictive value for concurrent interstitial lung disease, while anti-TIF1-γ was best predictive of malignancies [10]. Anti-aminoacyl-tRNA synthetase (anti-ARS) antibodies are positive in 25–35% of patients with IIM, with anti-Jo-1 being the most common. This class of antibodies is most frequently associated with PM, interstitial lung diseases, and mechanic’s hands. Anti-Mi-2 antibodies are found in 10–30% of patients with IIM, typically DM. Also, anti-Mi-2 antibodies correlate with skin manifestations and lung sparing [11]. EMG reveals pathological spontaneous activity, which could be fibrillations, high-frequency discharges, or myotonic discharges. During voluntary muscle activity, owing to loss of myofibrils and contraction synchrony, patients with active myositis reveal small amplitude, short duration, and polyphasic motor unit potentials [12].

Adult-onset IIM, except for verified cases of IBM, is frequently associated with malignancies [1]. Inflammatory myopathies occur in 10–30% of all malignancies [3]. Solid-organ cancers: lung, gastrointestinal, breast, ovarian, pancreatic, and prostatic cancers are most frequently linked to inflammatory myositis. Histologically, adenocarcinomas account for almost 70% of myositis associated with neoplasia [13]. DM is the most frequent myositis associated with cancers. Results from the MYONET registry identified DM in 17% compared to 3% with antisynthetase syndrome, among those with cancer-associated myositis [14]. Hematological malignancies are less frequently associated with inflammatory myopathies, and those that are most frequently reported are the B-cell, non-Hodgkin’s lymphomas (NHL). In a single-center retrospective cohort study by Mecoli et al., IIM patients reported a standardized prevalence ratio of 2.71 for lymphoma overall and 3.91 for DM [15]. Follicular lymphoma, a type of B-cell NHL, is rarely associated with autoimmune or paraneoplastic syndromes. A literature review identified only a few cases of follicular lymphoma-associated myositis published to date [2,3,16].

Molecular mimicry between tumor and muscle antigens may lead to cross-reactive immune responses. Tumor neoantigens or stress-induced overexpression of these proteins can trigger both antitumor activity and myositis. The PD-1/PD-L1 pathway is dysregulated: elevated PD-L1 appears paradoxically protective in myositis, but persistent immune activation may overwhelm this effect, permitting both cancer growth and muscle inflammation [7].

Cancer-associated myositis typically demonstrates a temporal relationship with the neoplasm, resistance to standard therapies, improvement with cancer therapy/remission, and relapse with cancer recurrence [17]. Many studies classify inflammatory myopathy as cancer-associated myositis when cancer is diagnosed within three years of myositis diagnosis [18]. A 2018 study reported a median interval between diagnosis of malignancy and myositis as 1 month [19]. Zhou et al., in China, reported a median of 7.2 months between malignancy and myositis with an interquartile range of 0 to 20.4 months [20]. New onset inflammatory myopathy after the age of 40 years, dysphagia, DM, severe skin involvement, persistent disease activity despite immunosuppressive therapy, and anti-TIF1- or anti-NXP2 γ positivity are considered high risk for IIM related to cancer [1]. Except for patients with juvenile onset IIM or confirmed inclusion body myositis, all others with IIM are recommended to undergo basic cancer screening at the time of IIM diagnosis. In those with a high risk, a more comprehensive cancer screening is recommended [1].

Treatment of IIM includes addressing the underlying cause and using immunosuppression. Steroids are often considered the first-line agent. Methotrexate or azathioprine is frequently used as a steroid-sparing agent. Alternate agents, with limited data, include intravenous immunoglobulin, mycophenolate mofetil, rituximab, alemtuzumab, and cyclophosphamide [21]. Patients with cancer-associated myositis often respond to cancer chemotherapy and have a response correlated with tumor treatment response.

Our patient presented with proximal muscle weakness, dysphagia, and elevated muscle injury markers consistent with myositis, further supported by the radiological evidence and EMG pattern. She was positive for anti-Mi-2α and anti-EJ antibodies. Anti-Mi-2α is strongly associated with DM with a high positive predictive value and specificity [22]. However, the patient had isolated muscle involvement with no cutaneous lesions. Anti-EJ antibodies are anti-ARS autoantibodies typically associated with antisynthetase syndrome. Antisynthetase syndrome is characterized by myositis, interstitial lung disease, arthritis, and, less commonly, rashes, Raynaud’s phenomenon, and fever. It is considered a distinct subtype of IIM by some authors [23]. Anti-ARS are also frequent in PM, but anti-Jo-1 is the most frequent [24]. The concomitant presence of anti-Mi-2α and anti-EJ suggests the likelihood of an overlap syndrome, rather than an isolated pathology. She was confirmed to have NHL—follicular lymphoma on lymph node biopsy. Inflammatory myopathies associated with hematological malignancies are rare. Her myopathy symptoms responded well to immune modulation with methotrexate; however, her muscle enzymes, through a shown response, failed to normalize. She was hesitant to undergo lymphoma chemotherapy and ultimately decided to proceed with isolated monoclonal anti-CD20 therapy. Initiation of monoclonal antibodies further improved her CPK levels; however, she failed to complete the regimen and was lost to follow-up. In summary, this case depicts a probable paraneoplastic-triggered overlap myositis: a unique combination of follicular lymphoma with overlap myositis, PM, antisynthetase syndrome, or DM sine dermatitis.

Limitation: The lack of a muscle biopsy limits confirmation of the diagnosis. However, the combination of clinical features, imaging, and electrodiagnostic studies was highly suggestive of an inflammatory myopathy. As the patient was lost to follow-up, we are not able to provide the long-duration response/results to therapeutic interventions.

## 4. Conclusions

Inflammatory myopathies associated with follicular lymphoma are sporadic and diagnostically challenging. Adult-onset inflammatory myopathy should prompt screening for cancers. Clinicians should maintain a high index of suspicion for occult malignancies, including hematological neoplasia in patients with inflammatory myopathies.

## Figures and Tables

**Table 1:** Hematologic and biochemical parameters at presentation.

Parameter	Results				Reference Range
	Visit 1	Visit 2	Visit 3	Visit 4	
Hemoglobin	10.6	10.1	9.2	9.9	12–16 g/dL
Leukocyte count	5.8	7.9	5.6	4.9	4.8–10.8 k/μL
Platelets	358	404	308	256	150–400 k/μL
Sodium	143	139	142	144	135–145 mEq/L
Potassium	4.1	3.5	4.9	4.0	3.5–5 mEq/L
Calcium	9.5	8.9	9.1	9.0	8.5–10.5 mg/dL
Magnesium	1.9	2.2	2.5	2.1	1.5–2.7 mg/dL
Phosphate	4.8	4.3	3.7	3.9	2.5–4.5 mg/dL
BUN	10	11	15	12	7–20 mg/dL
Creatinine	0.8	0.9	0.8	0.8	0.5–1.5 mg/dL
Bilirubin Total	0.3	0.3	0.5	0.4	0.2–11 mg/dL
Bilirubin Direct	0.1	0.2	0.2	0.1	0–0.3 mg/dL
AST	402	248	165	76	9–36 U/L
ALT	138	76	53	54	5–40 U/L
ALP	38	47	86	73	43–160 U/L
GGT	17	28	--^#^	--^#^	3–65 U/L
Albumin	4.3	3.9	3.6	4.0	3.5–5.5 g/dL
CRP	18.2	14.7	10.9	10.2	<5 mg/dL
LDH	912	846	375	219	100–190 U/L
CPK	10510	8640	1085	452	20–200 U/L
Aldolase	68.1	59.7	19.3	--^#^	<8.1/L
TSH	1.97	--^#^	--^#^	--^#^	0.4–4.5 mIU/L
HbA1c	8.7	--^#^	--^#^	--^#^	<5.7%

Visit 1: At presentation; Visit 2: Before initiation of methotrexate; Visit 3: Before initiation of Rituximab; Visit 4: Last follow-up visit with our institution; BUN: Blood urea nitrogen; AST: Aspartate aminotransferase; ALT: Alanine aminotransferase; ALP: Alkaline phosphatase; GGT: Gamma-glutamyl transferase; CRP: C-reactive protein; LDH: Lactate dehydrogenase; CPK: Creatine phosphokinase; TSH: Thyroid stimulating hormone; HbA1c: Hemoglobin A1c; --^#^ Result not available.

**Table 2:** Most frequent auto-antibodies in paraneoplastic inflammatory myopathies.

Autoantibody	Target Antigen	Myositis Type	Cancer Association
**Anti–TIF1-γ** **(p155/140)**	Transcription intermediar factor 1-gamma	DM (especially adult-onset)	Strong association with malignancy (breast, ovarian, GI, lung)
**Anti–NXP-2** **(anti–MJ)**	Nuclear matrix protein-2	Juvenile and adult DM	Moderate association with malignancy (especially in older males)
**Anti–Mi-2**	Chromatin remodeling helicase	Classic DM	Low malignancy risk
**Anti–MDA5** **(CADM-140)**	Melanoma differentiation associated gene 5	Clinically amyopathic DM	Variable (mainly Asian populations)
**Anti–SAE**	Small ubiquitin-like modifier activating enzyme	DM	Possible mild association
**Anti–Jo-1**	Histidyl-tRNA synthetase	Antisynthetase syndrome/PM	Rare
**Anti–PL-7** **Anti–PL-12** **Anti–EJ** **Anti–OJ**	Other aminoacyl-tRNA synthetases	Antisynthetase syndrome	Rare
**Anti–HMGCR**	3-hydroxy-3-methylglutaryl-coenzyme A reductase	IMNM	Low to moderate
**Anti–SRP**	Signal recognition particle	IMNM	Low
**Anti–PM/Scl**	Nucleolar exosome complex	Overlap myositis (scleroderma overlap)	Rare

DM: Dermatomyositis; PM: Polymyositis; IMNM: Immune-Mediated Necrotizing Myopathy; GI: Gastrointestinal.

## Data Availability

The original data presented in the study are included in the article, further inquiries can be directed to the corresponding author.

## Abbreviations

The following abbreviations are used in this manuscript:

IIM	Idiopathic inflammatory myopathies
CPK	Creatine phosphokinase
ANA	Antinuclear antibody
MRI	Magnetic resonance imaging
DM	Dermatomyositis
PM	Polymyositis
IMNM	Immune-mediated necrotizing myopathy
IBM	Inclusion body myositis
EMG	Electromyography
